# Safety and efficacy of S1P receptor modulators for the induction and maintenance phases in inflammatory bowel disease: A systematic review and meta-analysis of randomized controlled trials

**DOI:** 10.1097/MD.0000000000039372

**Published:** 2024-09-06

**Authors:** Abdullah Akram, Misha Ahmed, Kanza Farhan, Areeb Omer, Shamama Kaleem, Ali Tahir Khan, Uzma Aslam, Muhammad Abdullah Tahir, Saria Memon, Ayatul Karam, Humam Furqan, Muhammad Umair Anjum, Pratik Bhattarai

**Affiliations:** a Department of Medicine, Dow Medical College, Dow University of Health Sciences, Karachi, Pakistan; b Department of Medicine, Sindh Medical College, Jinnah Sindh Medical University, Karachi, Pakistan; c Department of Medicine, CMH Lahore Medical College & Institute of Dentistry, Lahore, Pakistan; d Department of Medicine, Fazaia Ruth Pfau Medical College, Karachi, Pakistan; e Department of Medicine, People’s University of Medical and Health Sciences for Women, Nawabshah, Pakistan; f Department of Medicine, Manipal College of Medical Sciences, Pokhara, Nepal.

**Keywords:** Crohn’s disease, inflammatory bowel disease, meta-analysis, S1P receptor modulator, ulcerative colitis

## Abstract

**Background:**

Inflammatory bowel disease (IBD) is a chronic inflammatory condition that significantly affects quality of life. Conventional treatments have had limited success. this study evaluates the safety and efficacy of Sphingosine 1-phosphate receptor modulators (S1PrMs) as a potential treatment for IBD.

**Methods:**

We conducted a thorough search of published literature on PubMed, EMBASE, and Google Scholar from 2000 to 2023. The inclusion criteria were randomized controlled trials (RCTs) with a target population comprising of IBD patients receiving either S1PrMs or placebo and a comparison of the 2. The statistical analysis was conducted using RevMan (version 5.4). Forest plots presented the results as risk ratios (RR) with a 95% confidence interval.

**Results:**

A total of 7 RCTs involving 2471 patients were included. The results were reported for both the induction and maintenance phases of treatment. in the induction phase, the intervention group proved to have a significantly higher incidence of histological remission (RR = 2.67; 95% CI [1.97, 3.60]; *P* < .00001), endoscopic improvement (RR = 2.06; 95% CI [1.66, 2.56]; *P* < .00001), clinical remission (RR = 2.23; 95% CI [1.43, 3.46]; *P* < .0004) and clinical response (RR = 1.37; 95% CI [1.01, 1.84]; *P* = .04) compared to the placebo group. Outcomes assessed in maintenance phase significantly favored the intervention group over placebo as well, histologic remission (RR = 2.39; 95% CI [1.83, 3.11]; *P* < .00001), endoscopic improvement (RR = 2.20; 95% CI [1.28, 3.77]; *P* = .004), clinical remission (RR = 3.03; 95% CI [1.84, 4.99]; *P* < .0001), and clinical response (RR = 1.74; 95% CI [1.25, 2.42]; *P* = .001).

**Conclusion:**

S1PrMs show promising potential for establishing histologic remission, endoscopic improvement, clinical remission, and corticosteroid-free clinical remission. With more studies and clinical trials, these modulators may become a reliable therapeutic choice for UC patients everywhere.

## 1. Introduction

Inflammatory bowel disease (IBD) is a chronic and recurrent inflammatory condition of the gastrointestinal tract, comprising of ulcerative colitis (UC) and Crohn’s disease. It is a global rising condition that not only affects the quality of human health but also imposes financial and economic burden on individuals and health systems worldwide. It has affected over 3 million people in the USA and Europe alone and its prevalence is estimated to exceed 0.3% in the many countries of Europe in the upcoming years.^[1]^

Conventional treatments, such as aminosalicylates, have shown moderate efficacy in mild to moderately affected patients but their effectiveness is limited in severe cases.^[2]^ Glucocorticoids due to their associated adverse effects are not recommended for long-term treatment.^[3,4]^ Other biologic drugs and Janus kinase inhibitors have also limited efficacy among all patients.^[5]^ Therefore, there is a need for more effective therapies for IBD patients with moderate to severe disease.^[6]^

Sphingosine 1-phosphate receptor modulators (S1PrMs) are emerging as a effective class of drugs for IBD. These compounds target the S1P receptor, a key regulator of inflammation and immune responses, offering a new approach to cope with the disease mechanism. The S1PrMs such as Ozanimod, Etrasimod, Mocravimod, and Amiselimod, have been effective in treating IBD, from moderate to severe patients and improving their health and lifestyle.^[6]^ Also, Ozanimod use has been approved in the US and European Union for the UC, a type of IBD, treatment. It shows high affinity binding to the subtypes of S1P receptors like S1P and S5P, proving it a potential therapeutic option for the various forms of IBD, including UC and Crohn’s disease.^[7]^

S1PrMs offer several advantages over conventional therapies. They have demonstrated efficacy in moderate to severe cases of IBD, where existing treatments have been less effective. Moreover, the early safety data from preclinical and phase 1 studies support their use as a potential long-term treatment option with improved tolerability.^[8]^ Also, the results from the induction and maintenance phases are favorable for the S1PrMs, the intervention group.^[7]^ The main purpose of this systemic review and meta-analysis is to evaluate the effectiveness, durability and safety of the S1PrMs in the treatment of moderate to severe cases of IBD from the results of recent RCTs.

## 2. Materials and methods

### 2.1. Data sources and search strategy

This meta-analysis was conducted conclusively with the Preferred Items for Systematic Reviews and Meta-Analysis (PRISMA) guidelines.^[9]^ Ethical Approval was not needed for this meta-analysis since it involved data collection from previously existing studies. A thorough electronic search encompassing 2000 through May 15, 2023, was performed on PubMed (Medline), EMBASE, and Google Scholar. The investigation was conducted by 2 impartial authors (MAB and ATK) without any restrictions or regard to language. On “clinicaltrials.gov,” we also looked for pertinent published or unpublished clinical trials. In addition, to find potentially relevant studies, we manually searched the reference lists of the included studies and related meta-analyses and review articles. An extensive list of words related to “inflammatory bowel disease,” “ulcerative colitis,” “Crohn’s disease,” “ozanimod,” “etrasimod,” “mocravimod,” and “ amiselimod” were searched. The detailed search strategy is given in the supplementary file (Table S1, Supplemental Digital Content, http://links.lww.com/MD/N445).

### 2.2. Study selection

Studies meeting the following criteria were included in this meta-analysis,

1. Study design should be a randomized controlled trial (RCT).
2. Target population comprising IBD patients, either ulcerative colitis or Crohn’s disease.
3. Comparison between any S1PR modulator and placebo.
4. Reporting of at least 1 safety or efficacy outcome.

The case reports, review articles, expert opinions, comments, cross-sectionals, editorials, and studies published before the year 2000 were excluded from the analysis.

### 2.3. Data extraction and assessment of study quality

Duplicate studies were deleted from the list after exporting the retrieved articles to Endnote Reference Library Software. The remaining articles were then carefully evaluated by the 2 independent reviewers (MA and AO), and only those articles matching the eligibility requirements were included. Based on the title and abstract, all articles were initially narrowed down. A third reviewer (SK) was involved in settling any discrepancies in the outcome. Data for the baseline characteristics and outcomes were taken from the finalized RCTs and entered into an online Microsoft Excel spreadsheet. Baseline characteristics included were age, BMI, gender, Mayo score, previous ulcerative colitis treatment, and baseline fecal calprotectin. The primary outcomes summarized in Table 1 and included in this meta-analysis are as follows: histological remission, endoscopic improvement, clinical remission, clinical response, serious adverse events, and worsening of ulcerative or ulcerative colitis flare-ups.

**Table 1:** Outcome table.

		Induction phase							Maintenance phase		
Study	Sample size	Histological remission, n (%)	Endoscopic improvement, n (%)	Clinical response, n (%)	Clinical remission, n (%)	Serious adverse events, n (%)	Worsening of ulcerative colitis, n (%)	Clinical remission, n (%)	Endoscopic improvement, n (%)	Histological remission, n (%)	Corticosteroid free clinical remission, n (%)
Sandborn 2021^[6]^	Placebo (n = 108)	8 (7.4)	12 (11.1)	28 (25.9)	6 (5.6)	3 (2.8)	N/A	42 (18.5)	60 (26.4)	37 (16.3)	38 (16.7)
	Ozanimod (1 mg; n = 429)	78 (18.2)	117 (27.3)	205 (47.8)	79 (18.4)	17 (4.0)	N/A	85 (37.0)	105 (45.7)	77 (33.5)	73 (31.7)
	Ozanimod (1 mg; n = 367)rc	64 (17.4)	100 (27.2)	193 (52.6)	77 (21.0)	23 (6.3)	N/A	N/A	N/A	N/A	N/A
Sandborn 2023 (UC 52 trial)^[10]^	Placebo (n = 144)	9 (6.3)	24 (16.7)	N/A	12 (8.3)	9 (6.3)	13 (9.0)	11 (7.6)	19 (13.2)	15 (10.4)	10 (6.9)
	Etrasimod (2 mg; n = 289)	66 (22.8)	108 (37.4)	N/A	81 (28.0)	20 (6.9)	22 (7.6)	94 (32.5)	113 (39.1)	79 (27.3)	94 (32.5)
Sandborn 2023 (UC 12 trial)^[10]^	Placebo (n = 116)	10 (8.6)	22 (19.0)	N/A	17 (14.7)	2 (1.7)	1 (0.9)	N/A	N/A	N/A	N/A
	Etrasimod (2 mg; n = 238)	41 (17.2)	78 (32.8)	N/A	62 (26.1)	6 (2.5)	9 (3.8)	N/A	N/A	N/A	N/A
Sandborn 2020^[11]^	Placebo (n = 27)	2 (8.0)	5 (17.8)	9 (32.5)	2 (8.1)	N/A	0	N/A	N/A	N/A	N/A
	Etrasimod (2 mg; n = 50)	8 (19.5)	21 (41.8)	26 (50.6)	16 (33.0)	N/A	2 (4.0)	N/A	N/A	N/A	N/A
	Etrasimod (1 mg; n = 52)	5 (10.2)	12 (22.5)	23 (43.7)	8 (16.0)	N/A	2 (3.8)	N/A	N/A	N/A	N/A
Sandborn 2016^[12]^	Placebo (n = 32)	3 (9.4)	N/A	12 (37.5)	2 (6.25)	3 (9.4)	2 (7.69)	4 (6.1)	N/A	5 (7.6)	N/A
	Ozanimod (0.5 mg; n = 65)	9 (13.84)	N/A	35 (53.84)	9 (13.84)	1 (1.53)	2 (3.07)	17 (26.1)	N/A	15 (23.0)	N/A
	Ozanimod (1 mg; n = 67)	15 (22.38)	N/A	38 (56.7)	11 (16.41)	3 (4.47)	3 (4.47)	14 (20.89)	N/A	21 (31.3)	N/A
Radeke 2020^[13]^	Placebo (n = 10)	N/A	N/A	3 (37.5)	0 (0)	N/A	N/A	N/A	N/A	N/A	N/A
	Mocravimod (1.2 mg; n = 17)	N/A	N/A	8 (57.1)	2 (14.28)	N/A	N/A	N/A	N/A	N/A	N/A
D’Haens 2022^[14]^	Placebo (n = 39)	N/A	N/A	20 (54.1)	15 (40.5)	1 (2.6)	N/A	N/A	N/A	N/A	N/A
	Amiselimod (0.4 mg; n = 37)	N/A	N/A	19 (48.7)	8 (28.2)	6 (15.4)	N/A	N/A	N/A	N/A	N/A

ATK employed the Cochrane Collaboration risk of bias method to evaluate the quality of included trials^[15]^ (Fig. S1A, B, Supplemental Digital Content, http://links.lww.com/MD/N445).

### 2.4. Statistical analysis

The statistical analysis was conducted using RevMan (version 5.4. Copenhagen: Nordic Cochrane Centre, The Cochrane Collaboration, 2014. For the visual presentation of the results, forest plots were computed. The random effects model presented the results as Risk ratios (RR) with a 95% confidence interval. To determine the unique effects of each study on a particular pooled outcome, the outcome was subjected to sensitivity analysis or meta-regression if the Higgin *I*^2^ value reported was >75%. A value of >75% was considered high for *I*^2^. A *P* value of .05 was considered significant throughout the analyses.

### 2.5. Publication bias

Publication bias was analyzed via Egger’s test using Comprehensive Meta-Analysis version 3.0 (Biostat, Inc., Englewood). A *P* value >.05 indicated no publication bias in our outcomes (Table 2). Furthermore, funnel plots were computed to assess the publication bias visually, and a symmetrical distribution of studies along the vertical axis also demonstrated the absence of publication bias. Funnel plots are provided in the supplementary file (Fig. S3A–F, Supplemental Digital Content, http://links.lww.com/MD/N445).

**Table 2:** Eggers’s test.

Outcomes	*P* value
Clinical response	.65
Endoscopic improvement	.53
Histological remission	.47
Serious adverse events	.85
Clinical remission	.93
Worsening of ulcerative colitis	.39

## 3. Results

### 3.1. Study selection

**Figure 1.**
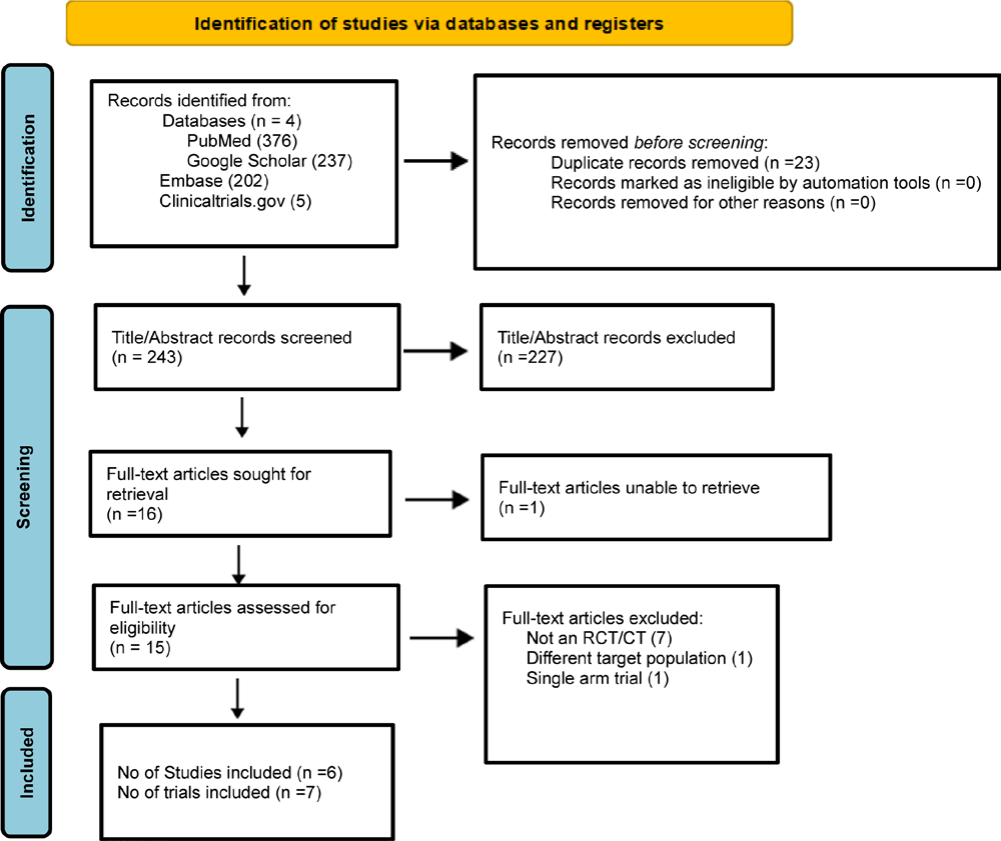
PRISMA 2020 flow diagram for new systematic reviews, which included searches of databases and registers only.

Extensive research was conducted on various electronic databases: PubMed (4 articles), Google Scholar (237 articles), Embase (202 articles), and ClinicalTrials.gov (5 articles) from 2000 onwards which yielded 243 results after screening. Two independent reviewers screened these articles. Initially, 243 articles were selected based on their titles and abstracts. Considering eligibility criteria, 227 articles were excluded after reviewing the titles and abstracts. Subsequently, the full texts of 16 articles were retrieved for further evaluation. Fifteen of these articles were deemed eligible. Ultimately, a total of 6 studies, 7 randomized control trials ^[6, 12, 10,11,13,14]^, that met the inclusion criteria were included in our meta-analysis. The Prisma flow diagram, as illustrated in Figure 1, guides us through the steps of identification, screening, and selection of articles that meet the study’s criteria.

### 3.2. Patients’ demographics

The 7 RCTs selected for the statistical analysis comprised 2471 patients (860 in the placebo group, 1611 in the intervention group). The mean age of the patients ranged between 31 and 43 years. Table 3 provides information on baseline characteristics which include: BMI, age, Mayo score, previous treatment for ulcerative colitis, and baseline fecal protein. Table 1 summarizes the 6 primary outcomes which includes: histologic remission, endoscopic improvement, clinical remission, clinical response, serious adverse events, worsening of ulcerative colitis, or ulcerative colitis flare-ups. The details like inclusion/exclusion criteria, primary outcomes, follow-ups, and treatments used in each trial were also reported.

**Table 3:** Baseline characteristics.

Study		Age	Male	BMI	Total Mayo score	Previous ulcerative colitis treatment					Baseline fetal protein
						Steroids	5-ASA	TNFα antagonists	AIA	Anti-IL 12/23 antibodies	
Sandborn 2023 (UC 52 trial)^[10]^	Placebo (n = 114)	38.9 (14.0)	88	25.4 (5.5)	9.0 (1.4)	101	95	31	19	1	NA
	Etrasimod 2 mg (n = 289)	41.2 (14.0)	152	24.4 (5.5)	9.0 (1.5)	224	197	60	28	6	NA
Sandborn 2023 (UC 12 trial)^[10]^	Placebo (n = 116)	40.4 (13.3)	73	25.4 (5.5)	8.8 (1.5)	98	85	29	10	4	NA
	Etrasimod 2 mg (n = 238)	40.3 (13.5)	135	24.4 (5.5)	8.7 (1.5)	117	149	57	33	5	NA
Sandborn 2021^[16]^	Placebo (n = 216)	41.9 (13.6)	143 (66)	25.1 (4.5)	8.9 (1.4)	162	210	65	NA	NA	NA
	Ozanimod (n = 429)	41.4 (13.5)	245 (57)	25.4 (5.5)	8.9 (1.5)	322	418	130	NA	NA	399–2532
	Ozanimod (n = 367)	42.1 (13.7)	214 (58)	25.9 (5.8)	9.1 (1.5)	286	362	159	NA	NA	421–2881
Sandborn 2020^[11]^	Placebo (n = 54)	44.8 (14.85)	32 (59)	25.8 (4.6)	NA	16	53	18	12	NA	NA
	Etrasimod 1 mg (n = 52)	43.2 (12)	30 (58)	24.9 (3.6)	NA	13	49	15	4	NA	30–24,190
	Etrasimod 2 mg (n = 50)	40.4 (12)	27 (54)	24.0 (5.5)	NA	18	46	17	7	NA	71–21,559
Radeke 2020^[13]^	Placebo (n = 10)	31.7 (9)	4	25.34 (5.76)	NA	10	NA	NA	NA	NA	NA
	KRP203 1.2 mg (n = 17)	40.1 (16)	11	23.452 (3.20)	NA	15	NA	NA	NA	NA	NA
D’Haens 2022^[14]^	Placebo (n = 37)	31 (20–56)	64.9%	22.8 (5.02)	NA	20	13	24	0	NA	NA
	Amiselimod (n = 39)	34 (20–58)	59%	22.62 (4.0)	NA	17	12	22	8	NA	2725.7 [4507.6]
Sandborn 2016^[12]^	Placebo (n = 65)	41.9 (12)	35 (54)	NA	8.6 (1.5)	24	57 (88)	10 (15)	NA	17 (26)	NA
	Ozanimod 0.5 mg (N = 65)	38.8 (12)	32 (49)	NA	8.3 (1.5)	22	53 (82)	13 (20)	NA	24 (37)	66–11,108
	Ozanimod 1.0 mg (N = 67)	41.8 (11)	48 (72)	NA	8.5 (1.6)	27	53 (79)	13 (19)	NA	22 (33)	10–10,511

### 3.3. Clinical remission

All the included studies reported Clinical remission as an outcome measure in the induction phase. There was more incidence of clinical remission in the S1PrM group as compared to the placebo group, and the results are statistically significant (RR = 2.23; 95% CI [1.43, 3.46]; *P* < .0004; *I*^2^ = 55%).

**Figure 2.**
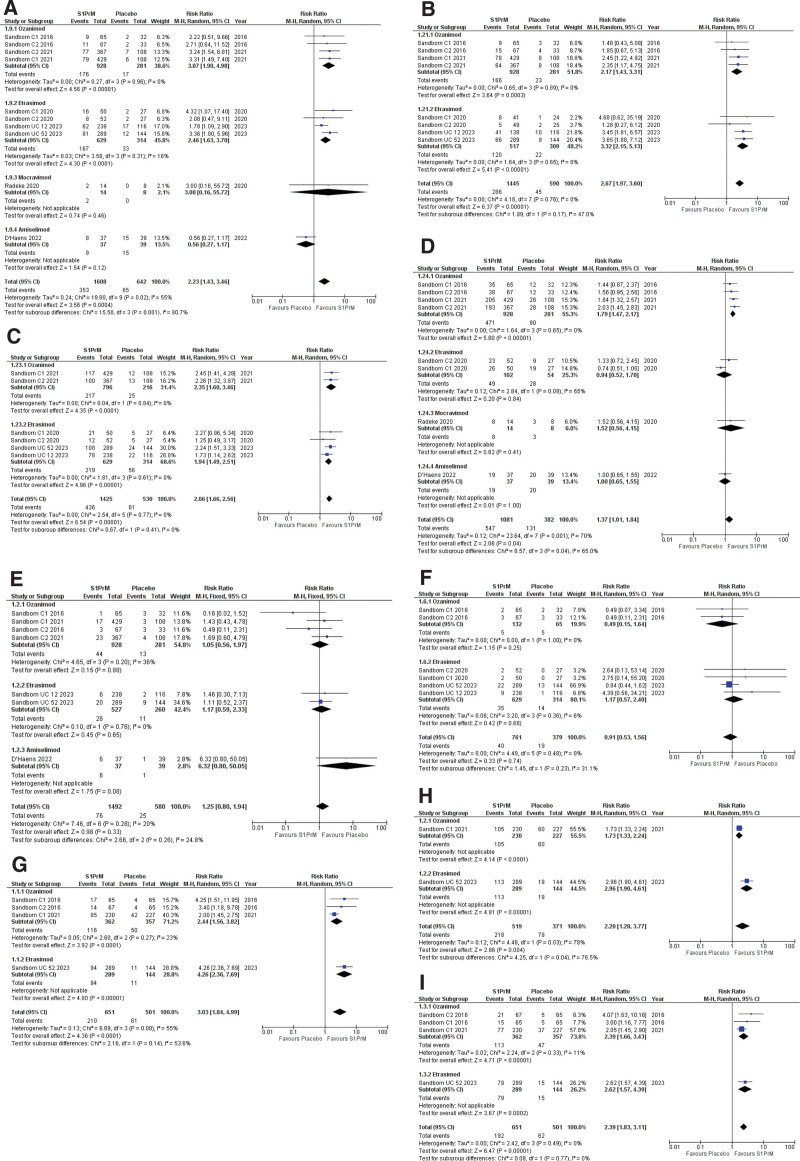
(A) Forest plot of clinical remission during the induction phase. (B) Forest plot of histological remission during the induction phase. (C) Forest plot of endoscopic improvement during the induction phase. (D) Forest plot of clinical response during the induction phase. (E) Forest plot of serious adverse events during the induction phase. (F) Forest plot of worsening ulcerative or ulcerative colitis flare-ups during the induction phase. (G) Forest plot of clinical remission during the maintenance phase. (H) Forest plot of endoscopic improvement during the maintenance phase. (I) Forest plot of histological remission during the maintenance phase. CI = confidence interval.

Furthermore, subgroup analysis during the induction phase revealed that ozanimod (RR = 3.07; 95% CI [1.90, 4.98]; *P* < .00001; *I*^2^ = 0%) and etrasimod (RR = 2.46,; 95% CI [1.63, 3.70]; *P* < .0001; *I*^2^ = 16%) reported a high incidence of clinical remission as compared to the placebo group and the results were statistically significant. However, mocravimod (RR = 3.00; 95% CI [0.16, 55.72]; *P* = .46; *I*^2^ = 0%) and amiselimod (RR = 0.56; 95% CI [0.27, 1.17]; *P* = .12; *I*^2^ = 0%) did not show any significant difference when compared to placebo. The results of the subgroup analysis were statistically significant (*P* = .001; *I*^2^ = 80.7%; Fig. 2A).

Clinical remission was reported as an outcome measure in the maintenance phase in 2 out of 7 studies. The forest plot favored the S1PrM group as compared to the placebo group, and the results were statistically significant (RR = 3.03; 95% CI [1.84, 4.99]; *P* < .0001; *I*^2^ = 55%). Moderately heterogeneity was reported in these results.

Based on the drug type of S1PrM, subgroup analysis evaluated that the incidence of clinical remission is significantly greater in ozanimod (RR = 2.44; 95% CI [1.56, 3.82]; *P* < .0001, *I*^2^ = 23%) and etrasimod (RR = 4.26; 95% CI [2.36, 7.69]; *P* < .00001) as compared to the placebo. The overall results for subgroup analyses were not significant (*P* = .14; *I*^2^ = 53.6%; Fig. 2G).

### 3.4. Histologic remission

Histologic remission was reported in 4 out of 7 studies during the induction phase and 3 out of 7 during the maintenance phase. During the induction phase, the forest plot reported that histological remission was significantly more in the intervention group than in the placebo group. (RR = 2.67; 95% CI [1.97, 3.60]; *P* < .00001; *I*^2^ = 0%). Based on the type of S1PrM drug, subgroup analysis revealed that Ozanimod (RR = 2.17; 95% CI [1.44, 3.31]; *P* = .0003; *I*^2^ = 0%) and Etrasimod (RR = 3.32; 95% CI [2.15, 5.13]; *P* < .00001; *I*^2^ = 0%) favored the occurrence of histologic remission. Moderate heterogeneity was applicable to subgroup analysis (Fig. 2B).

The forest plot of the maintenance phase for histologic remission also favored intervention groups. Quantitative analysis of it demonstrated a significant difference (RR = 2.39; 95% CI [1.83, 3.11]; *P* < .00001, *I*^2^ = 0%). On subgroup analysis by type of Sphingosine-1-Phosphate receptor Modulator drug during the maintenance phase, ozanimod (RR = 2.39; 95% CI [1.66, 3.43]; *P* < .00001, *I*^2^ = 11%) and etrasimod (RR = 2.62; 95% CI [1.57, 4.39]; *P* = .0002) showed a significant occurrence of histological remission as compared to the placebo. There was no significant difference in the incidence of histological remission among different subgroups (*P* = .77, *I*^2^ = 0%; Fig. 2I).

### 3.5. Endoscopic improvement

All the included studies assessed the frequency of endoscopic improvement during the induction phase. The forest plot of the induction phase was inclined to the intervention group as compared to the placebo group. Quantitative analysis of the findings of studies illustrated a significant difference (RR = 2.06; 95% CI [1.66, 2.56]; *P* < .00001, *I*^2^ = 0%). Based on subgroup analysis, Ozanimod (RR = 2.35; 95% CI [1.60, 3.46] *P* < .0001; *I*^2^ = 0%) and Etrasimod (RR = 1.94; 95% CI [1.49, 2.51] *P* < .00001; *I*^2^ = 0%) reported a significant incidence of endoscopic remission compared to placebo (Fig. 2C).

Two studies assessed the frequency of endoscopic improvement during the maintenance phase. It favored the intervention group compared to placebo (RR = 2.20; 95% CI [1.28, 3.77]; *P* = .004; *I*^2^ = 78%). Very high heterogeneity was reported in these results. Furthermore, subgroup analysis during the maintenance phase reported that ozanimod (RR = 1.73; 95% CI [1.33, 2.24]; *P* < .0001) and etrasimod (RR = 2.96; 95% CI [1.90, 4.61]; *P* < .00001) revealed significant chance of endoscopic improvement than the placebo group comparatively. There was significant difference in the frequency of endoscopic improvement produced by subgroups (*P* = .04; *I*^2^ = 76.5%; Fig. 2H).

### 3.6. Clinical response

All studies included in the review assessed clinical response in the intervention and placebo group during the induction phase and 2 out of 7 studies reported this outcome in the maintenance phase. The forest plot of clinical response reported that it is significantly greater in the intervention group than in the placebo group. Quantitative analysis reported a significant difference (RR = 1.37; 95%CI [1.01, 1.84]; *P* = .04; *I*^2^ = 70%). Based on the type of S1PrM drug, subgroup analysis reported a significant occurrence of clinical response in Ozanimod (RR = 1.79; 95% CI [1.47, 2.17]; *P* < .00001; *I*^2^ = 0%) subgroup. However, no significant differences were observed in the Etrasimod (RR = 0.94; 95% CI [0.52, 1.70]; *P* = .84; *I*^2^ = 65%), Mocravimod (RR = 1.52; 95% CI [0.56, 4.15]; *P* = .41; *I*^2^ = 0%)and Amiselimod (RR = 1.00; 95% CI [0.65, 1.55]; *P* = 1.00; *I*^2^ = 0%) group when compared to placebo (Fig. 2D).

The forest plot of clinical response in maintenance phase also favored S1PrM as compared to placebo (RR = 1.74; 95% CI [1.25, 2.42]; *P* = .001; *I*^2^ = 48%; Fig. S2Q, Supplemental Digital Content, http://links.lww.com/MD/N445).

### 3.7. Serious adverse events

All studies showed evidence of serious adverse events as an outcome measure during the induction phase. Pooled analysis revealed that the incidence of serious adverse events is less in the placebo group than in the intervention group, but the results were not statistically significant (RR = 1.25; 95% CI [0.80, 1.94]; *P* = .33; *I*^2^ = 20%).

Based on the type of S1PrM drug, subgroup analysis presented that the risk of serious adverse events was greater in ozanimod (RR = 1.05 95% CI [0.56, 1.97]; *P* = .88; *I*^2^ = 36%), etrasimod (RR = 1.17; 95% CI [0.59, 2.33] *P* = .65; *I*^2^ = 0%), and amiselimod (RR = 6.32; 95% CI [0.80, 50.05]; *P* = .08) as compared to the placebo group but the results was not statistically significant. The overall results of subgroup analysis for the incidence of serious adverse events were not significant (*P* = .26; *I*^2^ = 24.8%; Fig. 2E).

### 3.8. Worsening of ulcerative colitis OR ulcerative colitis flare-ups

Four studies evaluated the worsening of ulcerative colitis between the placebo and intervention groups during the induction phase. Pooled analysis of these studies reported no significant difference in the worsening of ulcerative colitis between the placebo and intervention groups (RR = 0.91; 95% CI [0.53, 1.56]; *P* = .74, *I*^2^ = 0%).

On subgroup analysis by type of S1PrM drug, ozanimod (RR = 0.49; 95% CI [0.15, 1.64]; *P* = .25; *I*^2^ = 0%) and etrasimod group (RR = 1.17; 95% CI [0.57, 2.40]; *P* = .68; *I*^2^ = 6%) showed no significant difference than placebo comparatively. The overall results for subgroup analysis were not statistically significant (*P* = .23; *I*^2^ = 32.0%; Fig. 2F).

### 3.9. Secondary outcomes

The effect sizes of the secondary outcomes analyzed are given in Table 4. Symptomatic remission, endoscopic normalization, mucosal healing, and corticosteroid-free clinical significantly improved in the S1PrM group. However, there were more adverse events, including anemia and nasopharyngitis. Forest plots of secondary outcomes are provided in the supplementary file (Fig. S2A–P, Supplemental Digital Content, http://links.lww.com/MD/N445).

**Table 4:** Secondary outcomes.

Outcomes	Effect size (RR)	95% CI	*P* value
**Induction phase**			
**Symptomatic remission** (Etrasimod)	**1.84**	**1.44, 2.33**	**<.00001**
**Endoscopic normalization** (Etrasimod)	**10.72**	**6.31, 18.22**	**<.00001**
**Mucosal healing** (Ozanimod)	**2.85**	**1.73, 4.68**	**<.0001**
**Macular edema**	**0.80**	**0.17, 3.73**	**.78**
Ozanimod	0.82	0.09, 7.86	.86
Etrasimod	0.79	0.10, 6.37	.82
**Adverse events**	**1.12**	**1.02, 1.22**	**.01**
Ozanimod	1.04	0.88, 1.23	.67
Etrasimod	1.18	1.04, 1.33	.010
Mocravimod	0.98	0.75, 1.28	.89
Amiselimod	1.31	0.91, 1.87	.15
**AE leading to discontinuation of regimen**	**1.36**	**0.87, 2.14**	**.18**
Ozanimod	0.90	0.45, 1.78	.75
Etrasimod	1.84	0.89, 3.81	.10
Amiselimod	1.84	0.59, 5.79	.29
**Anemia**	**0.69**	**0.48, 0.99**	**.04**
Ozanimod	0.56	0.33, 0.96	.03
Etrasimod	0.85	0.52, 1.41	.54
Amiselimod	0.21	0.01, 4.24	.31
**Headache**	**1.38**	**0.83, 2.28**	**.21**
Ozanimod	1.05	0.43, 2.54	.91
Etrasimod	2.10	1.03, 4.31	.04
Amiselimod	0.70	0.22, 2.29	.56
**Nasopharyngitis**	**1.94**	**0.76, 4.97**	**.17**
Ozanimod	2.12	0.69, 6.48	.19
Amiselimod	1.58	0.28, 8.93	.60
**Nausea**	**1.72**	**0.69, 4.31**	**.24**
Ozanimod	0.73	0.12, 4.39	.73
Etrasimod	2.34	0.80, 6.80	.12
**Pyrexia**	**1.18**	**0.59, 2.37**	**.64**
Ozanimod	2.37	0.27, 20.42	.43
Etrasimod	1.21	0.56, 2.58	.63
Amiselimod	0.21	0.01, 4.24	.31
**Abdominal pain**	**0.93**	**0.44, 1.99**	**.86**
Ozanimod	0.79	0.10, 6.30	.83
Etrasimod	0.86	0.36, 2.05	.73
Amiselimod	2.11	0.20, 22.28	.54
**Arthralgia**	**1.15**	**0.60, 2.21**	**.68**
Ozanimod	1.17	0.41, 3.36	.77
Etrasimod	1.27	0.39, 4.12	.69
Amiselimod	0.70	0.12, 3.97	.69
**Bradycardia**	**2.09**	**0.46, 9.45**	**.34**
Ozanimod	1.63	0.20, 13.52	.65
Etrasimod	2.70	0.31, 23.29	.36
**Maintenance phase**			
**Mucosal healing** (Ozanimod)	**2.27**	**1.67, 3.09**	**<.00001**
**Corticosteroid free clinical remission**	**2.88**	**1.15, 7.20**	**.02**
Ozanimod	1.90	1.34, 2.68	.0003
Etrasimod	4.68	2.52, 8.71	<.00001

Outcomes are highlighted in bold.

## 4. Discussion

IBD, which affects millions of people worldwide, is a difficult condition to manage.^[17]^ The S1P receptor is a critical regulator of immunological responses and inflammation, and S1P receptor modulators target this receptor, providing a novel and promising method to modify the disease process.^[18]^ Ozanimod, etrasimod, mocravimod, and amiselimod have emerged as possible options among the S1P receptor modulators, igniting interest in IBD treatment.^[16,19,20]^ In the context of IBD, the findings of this meta-analysis provide insight into the relative efficacy of these selective S1P receptor modulators. We sought to evaluate the influence of these medications on various clinical outcomes throughout the induction and maintenance stages of treatment by analyzing the data from numerous randomized controlled trials (RCTs).

Seven randomized control trials were found to be eligible for inclusion in this meta-analysis after a thorough search of electronic databases. In the trials, the effects of S1P receptor modulators were studied in UC patients, and Crohn’s disease, and variables such as histologic remission, endoscopic improvement, clinical remission, clinical response, major adverse events, and the likelihood of ulcerative colitis worsening were assessed. To understand more about potential differences between these medicines, a subgroup analysis was performed based on the specific S1P receptor modulator used.

IBD medications include glucocorticoids, aminosalicylates, immunomodulators (thiopurines and methotrexate), as well as more recent biologic treatments like tumor necrosis factor (TNF) inhibitors, gut-selective integrin antagonists, and the recently approved interleukin-12 and −23 inhibitor, ustekinumab.^[21–23]^ However, a recent multicenter European cohort study assessing disease burden and unmet clinical requirements in persons with moderate-to-severe ulcerative colitis highlighted poor efficacy with conventional therapies.^[24]^ Many patients still do not respond to induction therapy (first non-responders) or lose their response over time (secondary responders) despite the significant increase in treatment alternatives over the past 20 years.^[25]^

According to researchers, new doors for treatment are opened because of Sphingosine-1-phosphate modulators that are approved for the treatment of different immune-mediated conditions.^[26,27]^ Clinical trials that targeted S1P receptors for inflammatory disorders were successful, and as a result, the non-selective S1P modulator fingolimod was approved for relapsing forms of multiple sclerosis. The basis for creating more selective S1P receptor modulators is provided by our study, which concentrates on selective S1P modulator receptors to reduce the likelihood of significant side effects.^[24]^ This meta-analysis is a valuable addition to evaluate the role of S1P receptor modulator in IBD as the literature is very scarce.

Ozanimod and Etrasimod are considered more effective S1P modulators for Ulcerative Colitis than Mocravimod and Amiselimod due to their efficacy, safety profiles, and mechanisms of action. They have demonstrated improved results in clinical trials, including lowering inflammation and improving mucosal repair, with potentially fewer adverse effects. Furthermore, they may have better pharmacokinetic qualities and target-specific mechanisms, making them more appropriate for the treatment of Ulcerative Colitis.^[25]^ In a phase II trial in patients with moderate-to-severe UC, a daily dose of 1 mg ozanimod resulted in a higher rate of clinical remission at 8 weeks, the primary endpoint, than placebo,^[12]^ and our study demonstrated that ozanimod and etrasimod significantly contributed to corticosteroid-free clinical remission. According to Lasa et al,^[28]^ ozanimod and infliximab performed superior to other biologics or small-molecule drugs in inducing clinical remission during the induction phase of UC. Dubinsky et al^[29]^ demonstrated that ozanimod performed comparable to ustekinumab but had more favorable infection adverse rates, supporting the finding of our study. Similarly, in an open-label extension trial by Vermeire et al,^[16]^ etrasimod significantly improved the outcomes of ulcerative colitis, including clinical response, clinical remission, and endoscopic improvement. However, in their study, 60% of the patients receiving etrasimod 2 mg reported treatment-emergent adverse events, with worsening ulcerative colitis and anemia being the most common. In our study, although adverse events were reported, the results were not statistically significant.

Our study had several strengths, including the following: Using strict eligibility criteria to choose studies for the meta-analysis improves the relevance and quality of the data included by ensuring that only studies that met specific inclusion and exclusion criteria were considered. All the RCTs included in our meta-analysis were high-quality and multicenter trials, enhancing our findings’ reliability. The internal validity of our results is strengthened by low heterogeneity in most of the outcomes. Our meta-analysis is a significant contribution that offers a novel viewpoint on IBD treatment because it focuses on selective S1P receptor modulators in the setting of ulcerative colitis. In both the induction and maintenance phases, subgroup analyses based on S1P receptor modulators (ozanimod, etrasimod, mocravimod, and amiselimod) enable a more nuanced understanding of the efficacy of these drugs individually, providing helpful insights to direct clinical decision-making.

### 4.1. Limitations

Acknowledging a few limitations in our study for a transparent approach is essential. There was some heterogeneity present in our outcomes, so we used random effects instead of fixed, which is used in 0% heterogeneity. The findings may be less generalizable because only RCTs were included. Secondly, some research failed to publish results during the maintenance phase, which might have affected the total results. Despite these drawbacks, the study offers insightful information that should be addressed in follow-up studies to add to the existing literature.

## 5. Conclusions

This meta-analysis, in conclusion, offers critical information about the use of selective S1P receptor modulators for treating IBD. S1PrMs show promising potential for establishing histologic remission, endoscopic improvement, clinical remission, and corticosteroid-free clinical remission. These findings are significant in improving IBD management and raising the prospect of better long-term results for disease management. With more studies and clinical trials, these modulators may become a reliable therapeutic choice for UC patients everywhere.

## Author contributions

**Conceptualization:** Abdullah Akram.

**Data curation:** Misha Ahmed, Areeb Omer, Shamama Kaleem, Humam Furqan.

**Formal analysis:** Misha Ahmed, Areeb Omer, Shamama Kaleem.

**Investigation:** Misha Ahmed, Kanza Farhan, Areeb Omer, Shamama Kaleem, Ali Tahir Khan, Muhammad Abdullah Tahir, Ayatul Karam, Humam Furqan.

**Methodology:** Abdullah Akram.

**Project administration:** Misha Ahmed.

**Resources:** Muhammad Umair Anjum, Pratik Bhattarai.

**Supervision:** Abdullah Akram, Muhammad Umair Anjum, Pratik Bhattarai.

**Validation:** Abdullah Akram.

**Visualization:** Ali Tahir Khan, Uzma Aslam, Muhammad Abdullah Tahir, Ayatul Karam.

**Writing – original draft:** Abdullah Akram, Misha Ahmed, Kanza Farhan, Uzma Aslam, Saria Memon.

**Writing – review & editing:** Abdullah Akram.

## Supplementary Material


