# Supplementary material for: Safety and efficacy of S1P receptor modulators for the induction and maintenance phases in inflammatory bowel disease: A systematic review and meta-analysis of randomized controlled trials
Source: Medicine (Baltimore). 2024 Sep 6;103(36):e39372. doi: 10.1097/MD.0000000000039372 (PMC11383267; doi:10.1097/MD.0000000000039372)

| **Supplementary Table 1: Search Strategy** |  |
| --- | --- |
| ( Fingolimod OR Gilenya OR Ozanimod OR RPC-1063 OR Siponimod OR BAF312 OR Ponesimod OR MT-1303 OR Etrasimod OR APD334 OR Sphingosine-1-phosphate receptor modulators OR S1PR agonists OR S1P receptor modulators OR S1P receptor agonists OR S1P receptor modulating drugs OR S1P modulators OR Sphingosine-1-phosphate modulators OR S1PRM OR Sphingosine 1-phosphate receptor modulating compounds OR S1PR inhibitors OR Sphingosine 1-phosphate receptor inhibitors OR S1P receptor inhibitors OR S1P receptor antagonists OR S1P antagonists OR S1PR antagonist drugs OR Sphingosine-1-phosphate receptor antagonist drugs OR Sphingosine-1-phosphate receptor signaling inhibitors OR Sphingomab OR Amiselimod OR KRP203 OR AJM300 OR CS-0777 OR ONO-4641 OR TY-52156 ) AND ( ulcerative colitis OR UC OR colitis ulcerosa OR idiopathic ulcerative colitis OR chronic ulcerative colitis OR ulcerative proctitis OR pancolitis OR inflammatory colitis OR Crohn's disease OR Crohn disease OR CD OR Crohn OR transmural colitis OR Crohn's colitis OR IBD OR inflammatory bowel disease ) |  |

**Supplementary material**

**Supplementary figure 1a:** Quality assessment of included randomized controlled trials using Cochrane risk of bias tool.


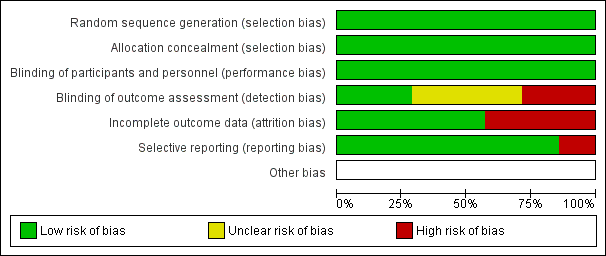


**Supplementary figure 1b:** Quality assessment of included randomized controlled trials using Cochrane risk of bias tool.


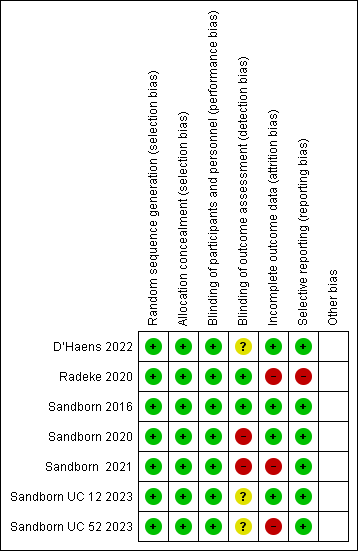


**Supplementary figure 2a:** Forest plot of symptomatic remission during induction phase.


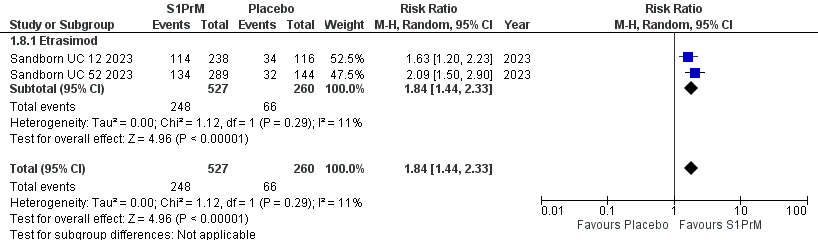


**Supplementary figure 2b:** Forest plot of endoscopic normalization during induction phase.


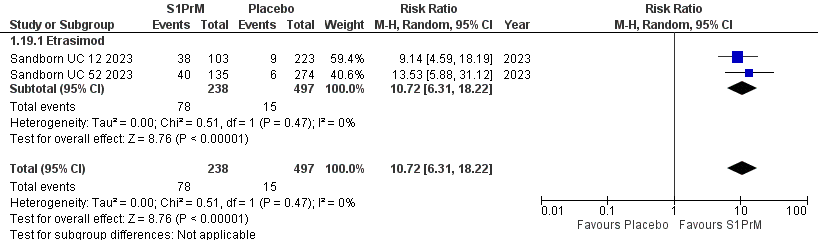


**Supplementary figure 2c:** Forest plot of mucosal healing during induction phase.


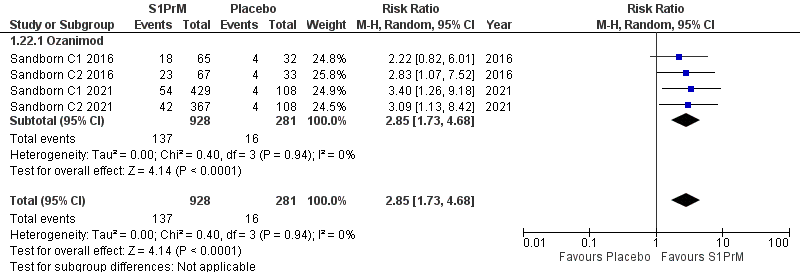


**Supplementary figure 2d:** Forest plot of macular edema incidence during induction phase.


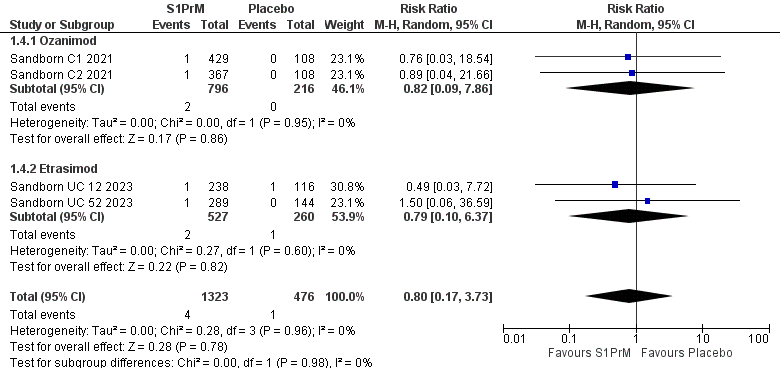


**Supplementary figure 2e:** Forest plot of adverse events incidence during induction phase.


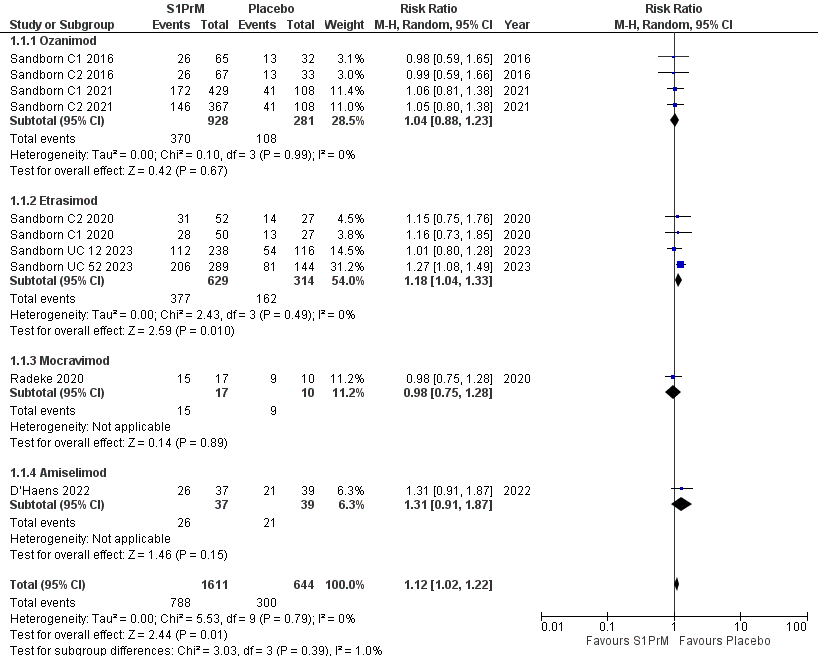


**Supplementary figure 2f:** Forest plot of adverse events leading to discontinuation of regimen during induction phase.


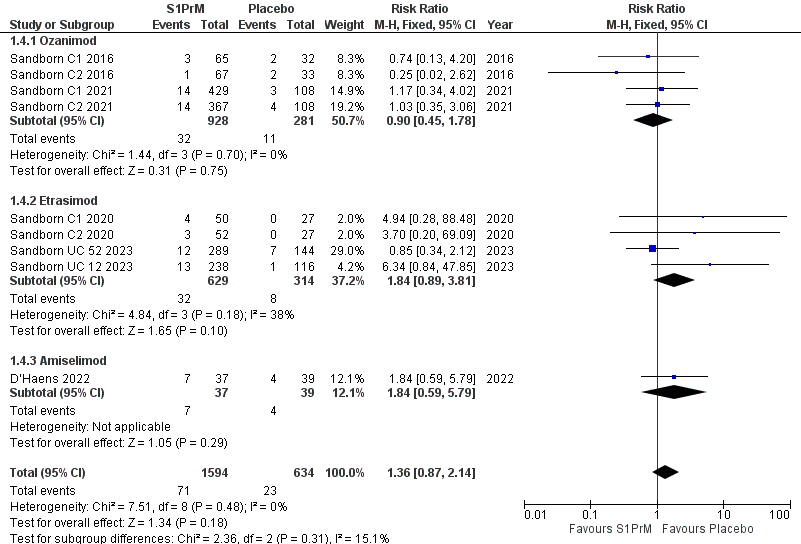


**Supplementary figure 2g:** Forest plot of anemia occurrence during induction phase.


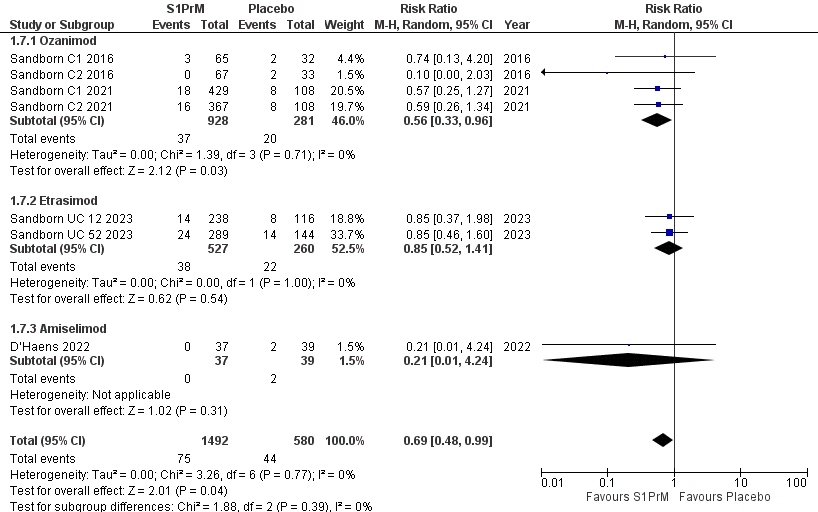


**Supplementary figure 2h:** Forest plot of headache incidence during induction phase.


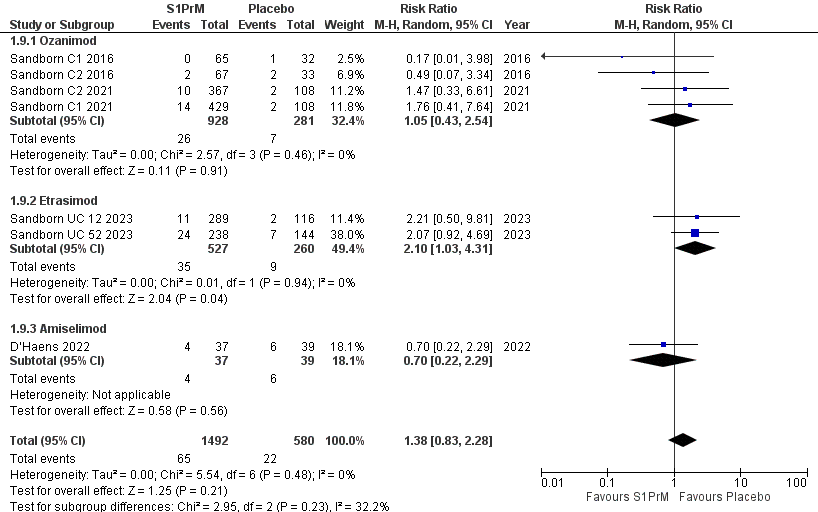


**Supplementary figure 2i:** Forest plot of nasopharyngitis incidence during induction phase.


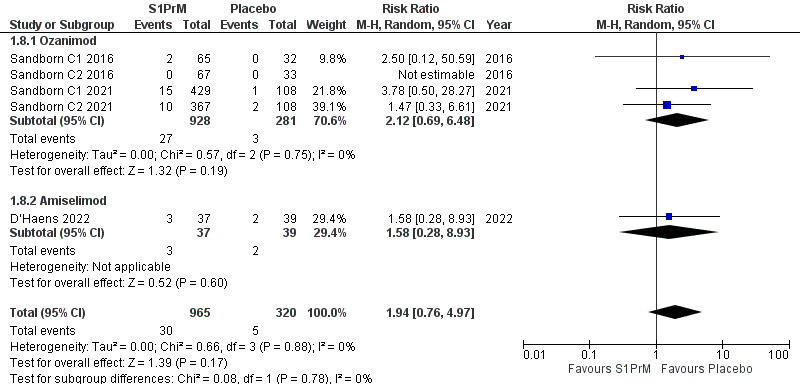


**Supplementary figure 2j:** Forest plot of nausea during induction phase.


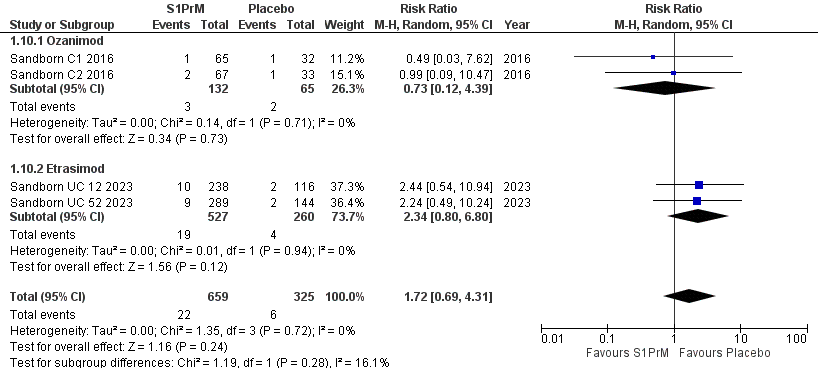


**Supplementary figure 2k:** Forest plot of pyrexia during induction phase.


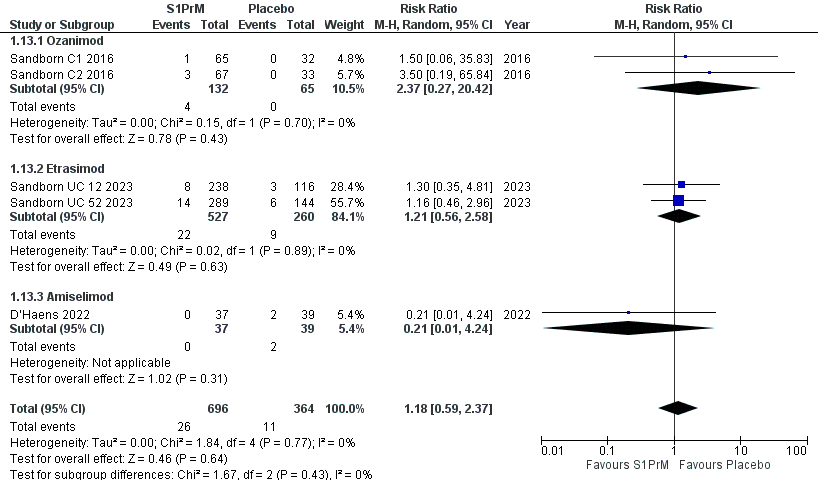


**Supplementary figure 2l:** Forest plot of abdominal pain during induction phase.


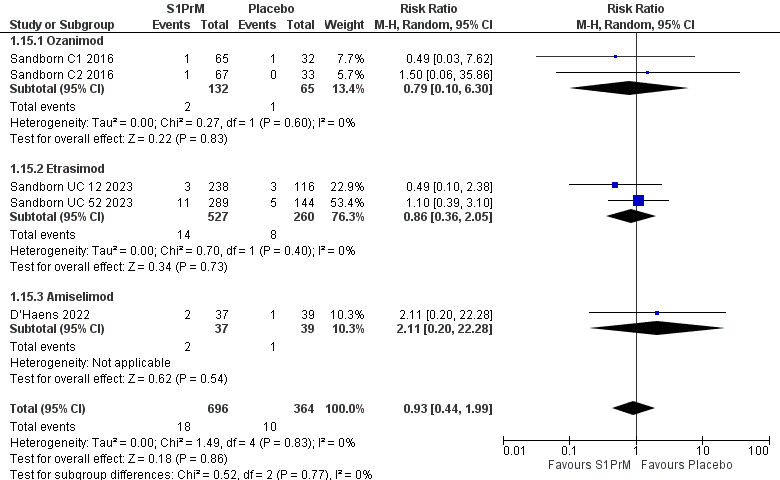


**Supplementary figure 2m:** Forest plot of arthralgia incidence during induction phase.


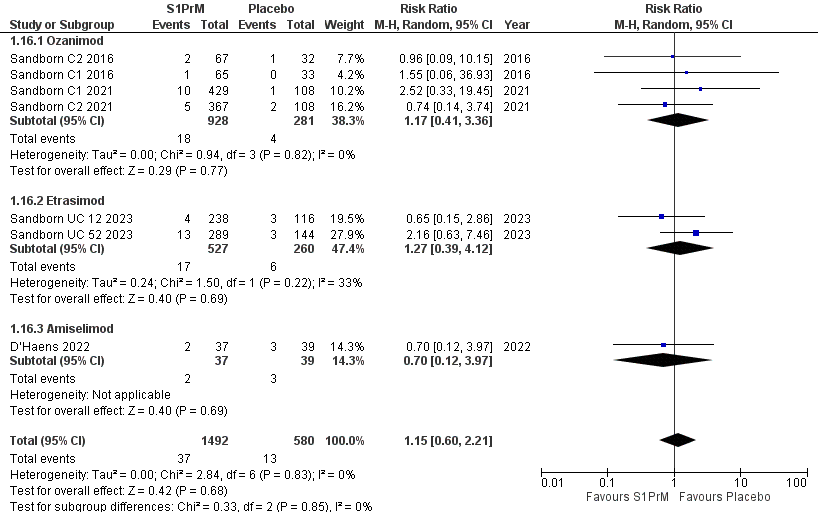


**Supplementary figure 2n:** Forest plot of bradycardia incidence during induction phase.


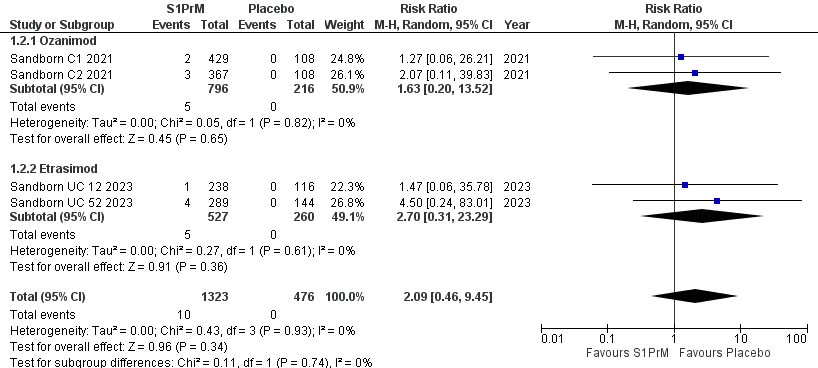


**Supplementary figure 2o:** Forest plot of corticosteroid-free clinical remission during maintenance phase.

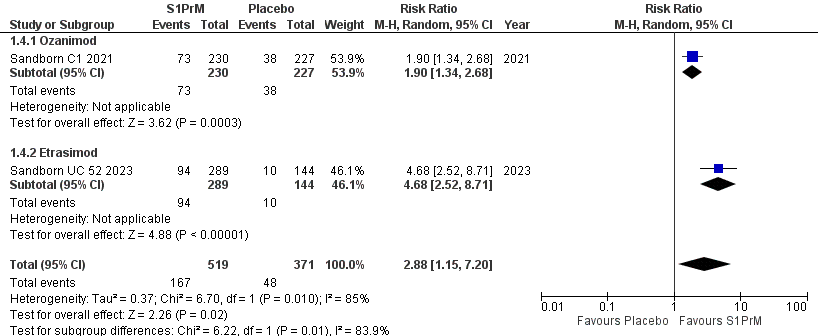


**Supplementary figure 2p:** Forest plot of mucosal healing during maintenance phase.


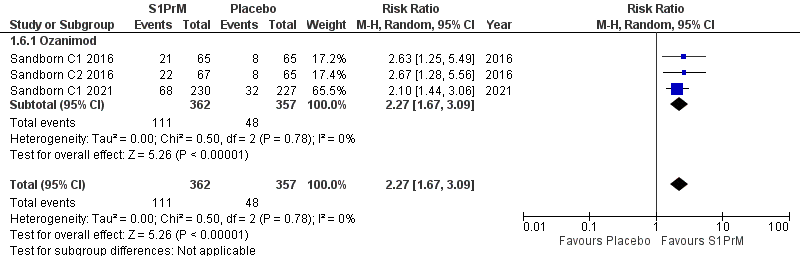


**Supplementary figure 2q:** Forest plot of clinical response during maintenance phase.


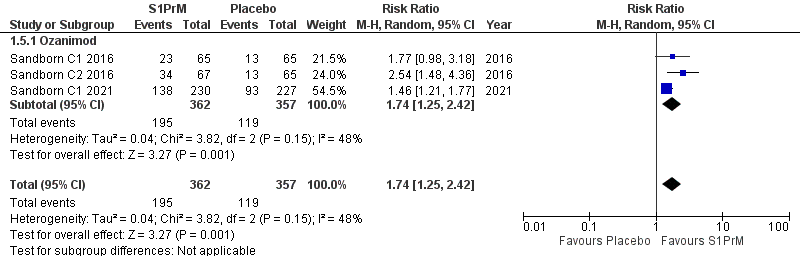


**Supplementary figure 3a:** Funnel plot of clinical response (induction phase).

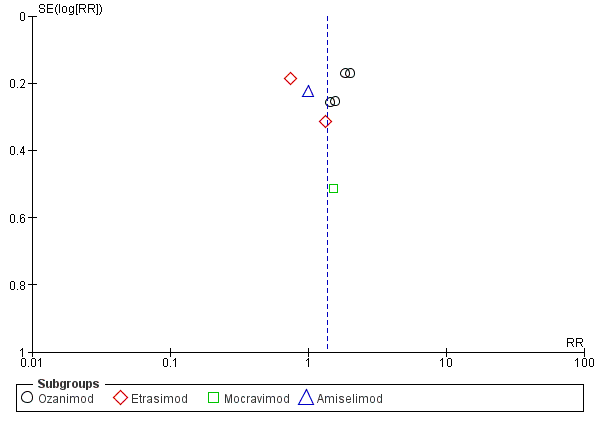


**Supplementary figure 3b:** Funnel plot of endoscopic improvement (induction phase).


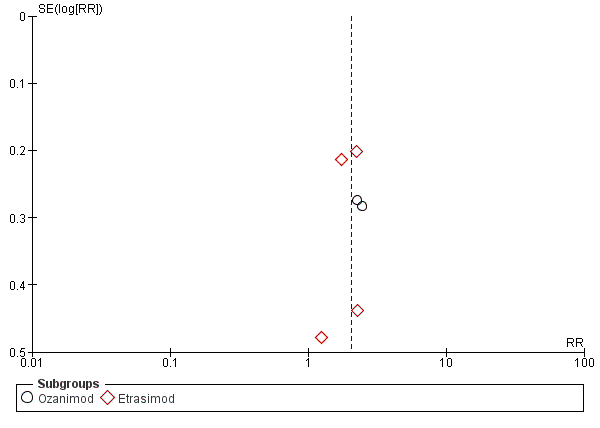


**Supplementary figure 3c:** Funnel plot of histological remission (induction phase).

**
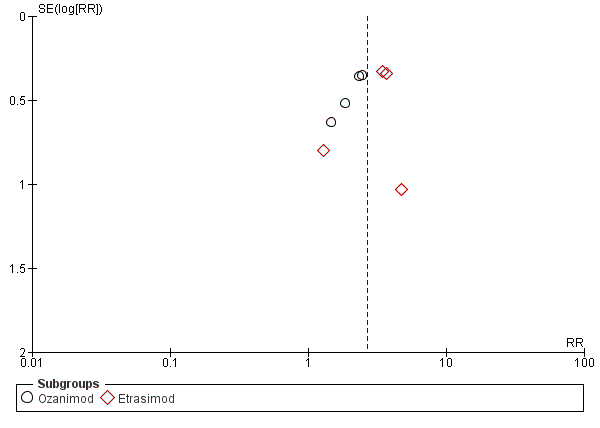
**

**Supplementary figure 3d:** Funnel plot of serious adverse events (induction phase).


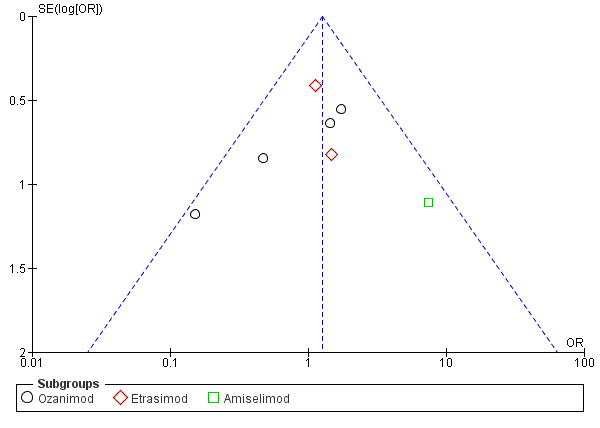


**Supplementary figure 3e:** Funnel plot of worsening ulcerative colitis or ulcerative colitis flare ups (induction phase)


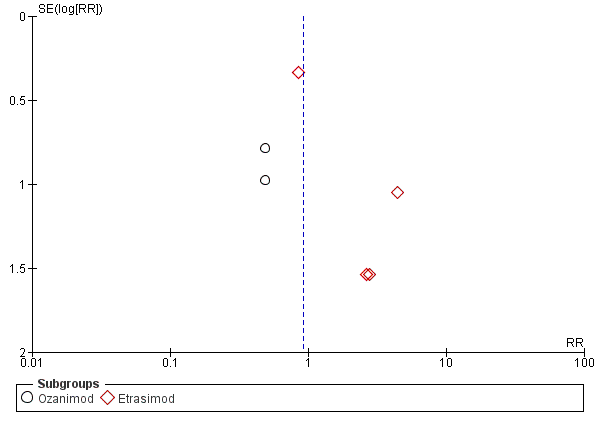


**Supplementary figure 3f:** Funnel plot of clinical remission (induction phase).


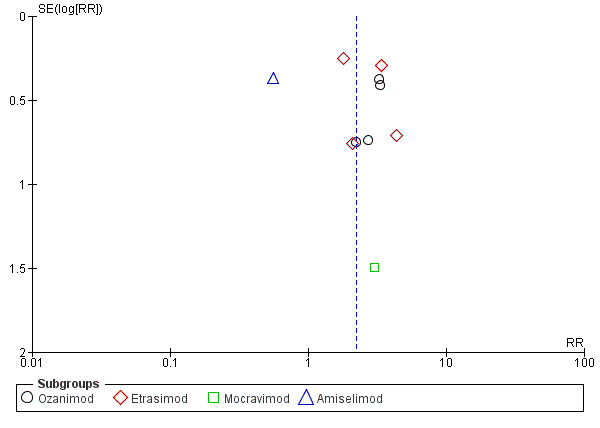

Supplement: Supplementary file 1 [file medi-103-e39372-s001.docx]
